# Antiviral Effect of Ribavirin against HCV Associated with Increased Frequency of G-to-A and C-to-U Transitions in Infectious Cell Culture Model

**DOI:** 10.1038/s41598-018-22620-2

**Published:** 2018-03-15

**Authors:** Andrea Galli, Helene Mens, Judith M. Gottwein, Jan Gerstoft, Jens Bukh

**Affiliations:** 1 Copenhagen Hepatitis C Program (CO-HEP), Department of Infectious Diseases and Clinical Research Centre, Hvidovre Hospital and Department of Immunology and Microbiology, Faculty of Health and Medical Sciences, University of Copenhagen, Copenhagen, Denmark; 2 Department of Infectious Diseases, Copenhagen University Hospital, Rigshospitalet, Copenhagen, Denmark; 3 Department of Clinical Medicine, Faculty of Health and Medical Sciences, University of Copenhagen, Copenhagen, Denmark

## Abstract

Ribavirin (RBV) is a broad-spectrum antiviral active against a wide range of RNA viruses. Despite having been used for decades in the treatment of chronic hepatitis C virus (HCV) infection, the precise mechanism of action of RBV is unknown. In other viruses, it inhibits propagation by increasing the rate of G-to-A and C-to-U transitions. Here, we utilized the J6/JFH1 HCV cell-culture system to investigate whether RBV inhibits HCV through the same mechanism. Infected Huh7.5 cells were treated with increasing concentrations of RBV or its phosphorylated forms. A fragment of the HCV NS5B-polymerase gene was amplified, cloned, and sequenced to estimate genetic distances. We confirm that the antiviral effect of all three RBV-drug forms on HCV relies on induction of specific transitions (G-to-A and C-to-U). These mutations lead to generation of non-infectious virions, reflected by decreased spread of HCV in cell culture despite relatively limited effect on virus genome titers. Moreover, treatment experiments conducted on a novel Huh7.5 cell line stably overexpressing adenosine kinase, a key enzyme for RBV activation, yielded comparable results. This study indicates that RBV action on HCV in hepatoma cell-culture is exerted through increase in mutagenesis, mediated by RBV triphosphate, and leading to production of non-infectious viruses.

## Introduction

Ribavirin (RBV; 1-(β-D-Ribofuranosyl)-1H-1,2,4-triazole-3-carboxamide) is a purine nucleoside analogue and currently the only licensed broad-spectrum antiviral. When introduced in the treatment of patients chronically infected with hepatitis C virus (HCV), a major cause of morbidity and mortality resulting from end-stage liver diseases, RBV in combination with pegylated α-interferon led to a major improvement in treatment outcome^1,2^. It subsequently remained a cornerstone of HCV treatment until the recent introduction of the highly-effective Directly-Acting Antivirals (DAAs)^3^. However, RBV is still a therapeutic option in combination with DAAs in special cases, such as presence of cirrhosis in genotype 3 infections, and in combination with Ombitasvir/Paritaprevir/r for genotype 1a and genotype 4^4–6^. Treatment with RBV is also recommended in severe cases of hepatitis E infection, severe respiratory syncytial virus (RSV) pneumonia, and hemorrhagic fevers such as Lassa- and Crimean-Congo-fever. In addition, RBV has proven efficacious *in vitro* against several RNA and DNA viruses^7^. Unfortunately, RBV has many adverse effects, also when administered without pegylated interferon-α mostly due to the induction of anemia^8^. Understanding its mode of action will hopefully facilitate the development of more potent broad-spectrum drug candidates with less adverse events.

Despite having been in use for several decades, the mechanism of action of RBV is not fully understood. In poliovirus^9,10^, lymphocytic choriomeningitis virus^11^, and foot-and-mouth disease virus^12^, RBV has been shown to act by increasing mutation rates by promoting G-to-A and C-to-U transitions. In addition, recent studies have demonstrated that RBV acted as a mutagen for different viruses^7,13^. As a mutagen, RBV structurally resembles adenosine or guanosine. Thus, when incorporated into nascent RNA as an analog of either adenine or guanine, it will pair equally well with either uracil or cytosine, thereby inducing G-to-A and C-to-U transitions. RNA viruses have extraordinary high mutation rates^14^, and it is hypothesized that RBV, by increasing the rate of G-to-A and C-to-U mutations, forces the virus over a mutational threshold resulting in the generation of non-infectious genomes (this mechanism of action is also referred to as lethal mutagenesis).

HCV infection is a leading cause of hepatic cirrhosis and hepatocellular carcinoma worldwide^15^. It is a positive-sense, single-stranded RNA virus, classified as a member of the *Hepacivirus* genus, family *Flaviviridae*^16,17^. The viral genome encodes three structural and seven nonstructural (NS) genes, translated as a single polyprotein which is then cleaved by cellular and viral proteases into individual proteins^16,17^. The viral RNA-dependent-RNA-polymerase is error-prone and lacks proofreading ability, leading to high variability and heterogeneity of HCV^18^. Phylogenetic analysis of HCV isolates led to the definition of seven major genotypes with more than 80 subtypes, which differ from one another by up to 30% at the nucleotide level^16,17,19^. Genetic variability also leads to the generation of *quasispecies* during infection, which can have an impact on treatment outcome^16,18,20^.

Several modes of action have been proposed for RBV in the control of HCV infection, including immune modulation, inhibition of the inosine monophosphate dehydrogenase (IMPDH)^21^, inhibition of the HCV RNA-dependent RNA polymerase^22^, and increase of mutation rate^23^. Previous studies on the mutagenic effect of RBV on treated HCV patients have shown conflicting results, with some authors reporting an association between antiviral effect and increased mutation frequency^24,25^, while others have not^26^. Similarly, *in vitro* studies have shown either increase^27,28^ or no increase^29^ in mutation frequencies upon RBV treatment, but these studies have been for the most part conducted in replicon systems, which do not recapitulate the entire viral life cycle. The only study performed on cell-culture adapted viruses could detect increased mutation frequencies but used a system that surprisingly led to clearance of infection under RBV treatment^28^.

To clarify the role of RBV as a mutagen in HCV infection, we tested its effect at different concentrations in an infectious HCV cell-culture model. The J6/JFH1 cell-culture system recapitulates the complete HCV life cycle, by replicating the full-length intra-genotypic J6/JFH1 genotype 2a recombinant^30,31^ in the human hepatoma-derived cell line Huh7.5^32^. We evaluated the effect on virus complexity by analyzing the mutational landscape of viral genomes obtained from supernatant in treated and untreated *in vitro* HCV infections. Our results show a correlation between the antiviral effect of RBV and the induction of predominantly G-to-A, as well as C-to-U transitions, supporting mutagenesis as one mechanism of action for RBV in the control of HCV infection.

## Results

### Ribavirin inhibits HCV spread and infectivity but has limited effect on virus RNA titers

**Figure 1:**
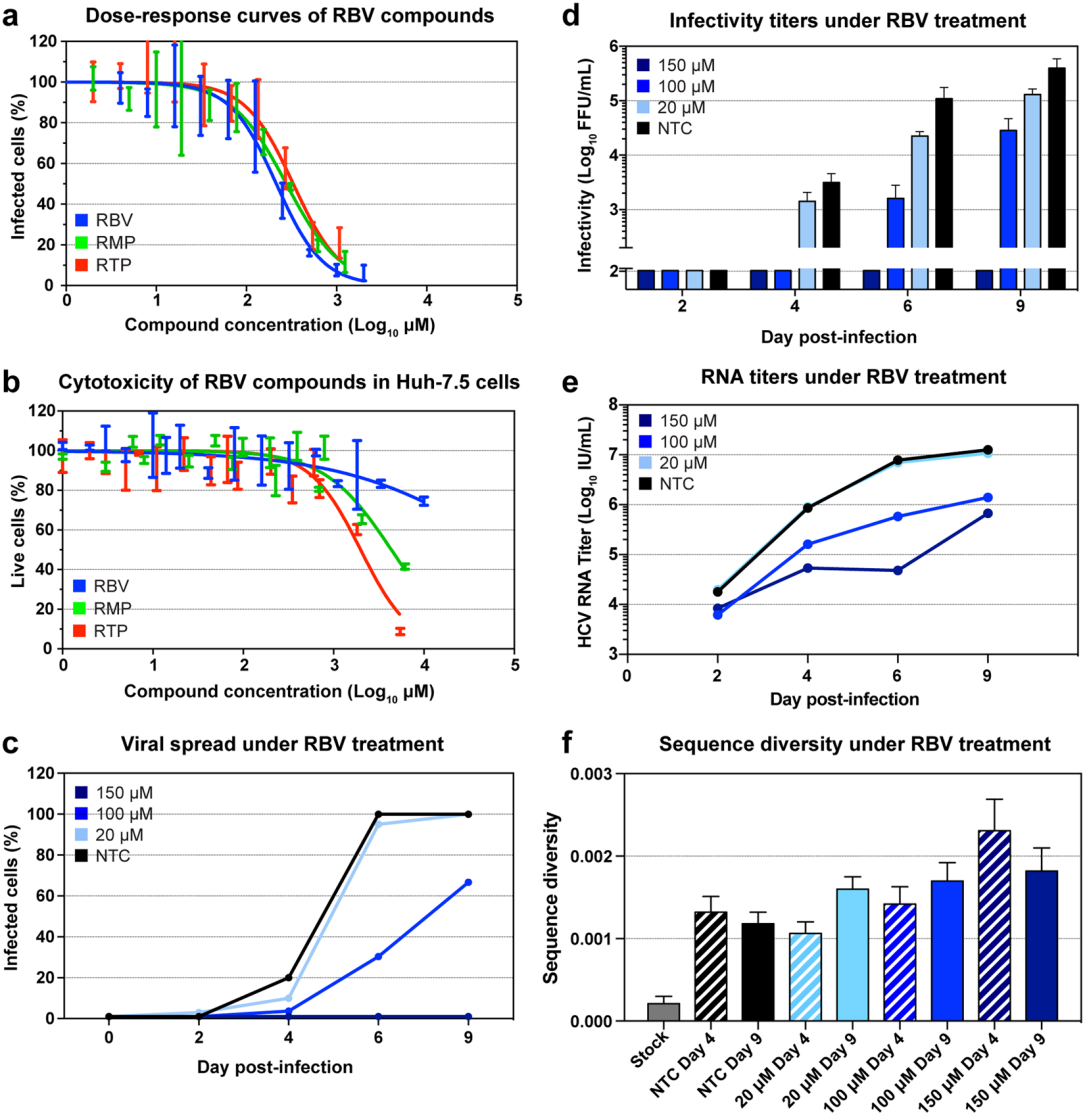
Ribavirin treatment of Huh7.5 cells infected with HCV J6/JFH1. (**a**) Dose-response curves of RBV, RMP and RTP in J6/JFH1 infected cells estimated by immunostaining of NS5A, obtained with triplicate determinations for each drug dilution. Data was normalized to non-treated controls, and curve fitting was thus performed with 0%-100% constraints. Error bars indicate standard deviation (SD). (**b**) Cell viability assay performed on Huh7.5 cells treated with RBV, RMP, and RTP. All data points were determined in triplicate; error bars indicate SD. (**c**–**f**) Panels representing data from one of two sets of independent experiments (for the second set of experiments see Supplementary Fig. 1). (**c**) Infection spread of J6/JFH1 virus estimated by immunostaining of NS5A in treated and non-treated samples. Data points represent the average of 1–3 determinations. (**d**) Viral infectivity of filtered supernatants obtained from treated and non-treated samples, data points represent the average of at least 3 determinations, error bars represent SD. (**e**) HCV RNA titers of viruses recovered from supernatants of treated and non-treated samples; data points represent averages of at least 2 measurements. (**f**) Nucleotide diversity of genomes extracted from stock virus and supernatants from RBV treated and non-treated samples, calculated as average p-distance. This distance is the average proportion of different nucleotides between sequence pairs. Sample sizes for each sample are found in Table 1. Banded bars indicate samples from day 4. Error bars represent standard deviations (SD).

To assess the effect of RBV on HCV infection in cell-culture, we treated Huh7.5 cells infected with the J6/JFH1 recombinant virus using increasing drug concentrations. Initially, we evaluated IC_50_ (50% inhibitory concentration) and cytotoxicity of RBV in this cell-culture model (Fig. 1a and b). Using a 48 hours dose-response assay, the IC_50_ of RBV on J6/JFH1 was estimated at 214 µM, whereas the cytotoxic effect of RBV on naïve cells was estimated to have an LC_50_ (50% lethal concentration) of 123 mM, with more than 90% live cells at concentrations of up to 1 mM. The concentration of RBV in hepatocytes of treated patients is unknown, but the concentration of RBV in human plasma and PBMC has been estimated to be 10–20 µM^33^. We previously observed that treatment of J6/JFH1 with 20 µM RBV had no effect on viral spread in cell culture^34^.

Based on these data and the IC_50_ estimation, we thus treated J6/JFH1-infected Huh7.5 cells with RBV concentrations of 20 µM, 100 µM, and 150 µM in parallel with non-treated control cells (NTC). Treatment was initiated 24 hours after infection and viral spread was subsequently monitored by immunostaining every 2–3 days. Viral supernatants collected at the same time points were used to determine HCV RNA and infectivity of released viruses. RBV inhibited virus spread and infectivity in cell culture in a concentration-dependent manner (Fig. 1 and Supplementary Fig. 1). Viral spread of J6/JFH1 was slightly reduced by 20 µM RBV, whereas higher concentrations delayed (100 µM) or inhibited (150 µM) viral spread (Fig. 1c). Viral infectivity was similarly reduced in a concentration-dependent manner, indicating that a reduction in the number of released infectious viral particles is the likely cause of spread inhibition (Fig. 1d). With the exception of cultures treated with 150 µM RBV, by day 9 all J6/JFH1 infections had spread to the majority of cultured cells and reached high infectivity levels. RBV reduced RNA titers of supernatant virus with increasing drug concentrations, although the effect was subtler compared to the effects on spread and infectivity (Fig. 1e). We observed growth of J6/JFH1 to RNA titers of 10^7^ IU/mL by day 9 in the NTC, as reported previously^35^. The RNA curves obtained from supernatants of treated cultures were reduced by 1–2 logs, depending on RBV concentration, and showed no rebound during the observation period. However, the reduction in viral infectivity was 1 to 2-fold higher than the decrease in RNA titers, for samples treated with 100 µM and 150 µM RBV. In particular, while RBV 150 µM led to full viral inhibition at day 9, showing a drop in infectivity of at least 3.5 logs, the supernatant viral RNA titers were only reduced by about 1.3 logs compared to the NTC, suggesting that virus RNA production was less affected. This is reflected in a sharp and significant drop in relative specific infectivity over time, indicating that the increase in HCV RNA titer is not matched by an equal increase in viral infectivity in samples treated with RBV 100 µM and 150 µM (Supplementary Fig. 2). Taken together, these results suggest the antiviral effect by RBV primarily involves impairing infectivity. Such pattern of viral spread would be expected, if RBV acted as a viral mutagen. To explore this hypothesis, we examined the mutational patterns of viruses obtained from cell culture supernatants.

### Increasing diversity of the virus population correlates with the antiviral effect of ribavirin

To study the mutational patterns of HCV under the effect of RBV, a 1245 base-pair region of the viral polymerase gene (NS5B) was amplified (positions 7847 to 9091 of J6/JFH1), cloned, and sequenced from virus recovered at day 4 and at day 9 post infection. An average of 62 sequences (range 32–81) per time point were analyzed (Table 1). In the optimization phase of the protocol, amplification was carried out using both a limiting dilution technique described by Mens *et al*.^36^ and standard molecular cloning. As had been reported previously^37^, we found no difference in sequencing depth or polymorphism detection between these approaches. Thus, molecular cloning was used for all subsequent data.

**Table 1:** Sequence analysis of supernatant J6/JFH1 from treated and untreated cultures.

Sample	Time	Drug	N^*a*^	π^*b*^	Diverg^*c*^	K^*d*^	G-to-A^*e*^	p-value^*f*^	C-to-U^*g*^	p-value^*h*^
*Day 4*										
NTC	d4		78	1.32	0.67	1.65	0.028		0.087	
RBV 20	d4	20 µM	81	1.06	0.55	1.32	0.046	○	0.044	○
RBV 100	d4	100 µM	53	1.42	0.71	1.76	0.076	●	0.057	○
RBV 150	d4	150 µM	32	2.31	1.23	2.88	0.214	●●●	0.145	●●●
RMP 20	d4	20 µM	48	0.90	0.45	1.12	0.039	○	0.045	○
RMP 100	d4	100 µM	38	1.80	0.91	2.24	0.065	○	0.100	○
RMP 150	d4	150 µM	32	2.67	1.38	3.32	0.136	●●●	0.213	●
RTP 20	d4	20 µM	39	1.64	0.85	2.04	0.047	○	0.069	○
RTP 100	d4	100 µM	30	1.80	0.91	2.24	0.072	○	0.127	○
ADK NTC	d4		37	2.75	1.48	3.42	0.109		0.022	
ADK 20	d4	20 µM	38	0.63	0.32	0.78	0.016	○	0.007	○
ADK 100	d4	100 µM	35	2.12	1.22	2.64	0.053	○	0.109	●
*Day 9*										
NTC	d9		78	1.18	0.60	1.47	0.036		0.045	
RBV 20	d9	20 µM	78	1.60	0.82	1.98	0.032	○	0.066	○
RBV 100	d9	100 µM	67	1.70	0.85	2.11	0.088	●	0.077	○
RBV 150	d9	150 µM	32	1.82	0.98	2.26	0.126	●●	0.068	○
RMP 20	d9	20 µM	41	2.19	1.10	2.72	0.076	●	0.086	○
RMP 100	d9	100 µM	40	1.68	0.85	2.09	0.078	●	0.068	○
RMP 150	d9	150 µM	31	1.98	1.01	2.46	0.150	●●●	0.096	○
RTP 20	d9	20 µM	33	2.07	1.05	2.58	0.047	○	0.091	○
RTP 100	d9	100 µM	30	1.82	0.91	2.26	0.062	○	0.145	●
RTP 150	d9	150 µM	37	0.93	1.15	1.16	0.025	○	0.265	●●●
ADK NTC	d9		30	1.64	0.86	2.05	0.051		0.018	
ADK 20	d9	20 µM	28	2.07	1.06	2.57	0.055	○	0.116	●
ADK 100	d9	100 µM	31	1.94	0.99	2.41	0.101	○	0.114	●

^*a*^Number of analyzed sequences.

^*b*^Diversity (nucleotide difference per site × 10^−3^).

^*c*^Divergence from J6/JFH1(nucleotide difference per site × 10^−3^).

^*d*^Average number of pairwise nucleotide differences.

^*e*^number of G-to-A changes per G in reference per sequence.

^*f*^p-value. Mann-Whitney test of the number of G-to-As compared to NTC.

^*g*^Number of C-to-U changes per C in reference per sequence.

^*h*^p-value. Mann-Whitney test of the number of C-to-Us compared to NTC.

^○^non-significant; ^●^p < 0.05; ^●●^p < 0.001; ^●●●^p < 0.0001.

Nucleotide diversity is a crude measure of the degree of polymorphism within a population. Natural selection has a u-shaped impact on virus diversity: highest diversities can be expected with intermediate selecstion pressure, whereas high selection pressure often leads to loss of diversity due to bottleneck events. However, if RBV acts as a mutagen, one would expect a linear correlation between selection and mutation. Nucleotide diversity among samples was estimated as p-distance with the Jukes and Cantor correction^38,39^ (Fig. 1f), calculated as the proportion of different nucleotides between each pair of sequences. The NTC displayed low nucleotide diversity at day 4 which did not increase at day 9 (1.3 × 10^–3^ vs 1.2 × 10^−3^ nucleotide differences per site, nds). In comparison to NTC, treatment with RBV resulted in a concentration-dependent increase in virus diversity, resulting in values of 1.6 × 10^−3^ nds (20 μM), 1.7 × 10^−3^ nds (100 μM), and 1.8 × 10^−3^ nds (150 μM) at day 9 (Fig. 1f). The stock virus used for inoculation was analyzed with the same protocol and showed a baseline diversity of 0.2 × 10^−3^ nds (n = 30). Nucleotide divergence is a measure of the degree of genetic distance between two populations. Nucleotide divergence from the J6/JFH1 reference strain, as well as between day 4 and day 9, was estimated as p-distance with Jukes and Cantor correction, computed as the average number of nucleotide substitutions per site between populations^40^. Treatment with RBV led to a time- and concentration-dependent increase in virus divergence from the reference J6/JFH1 sequence (Table 1). Analysis of samples from day 9 showed that NTC had a divergence from the J6/JFH1 reference strain of 0.6 × 10^−3^ nds. In comparison, treated samples had 0.8 × 10^−3^ nds (20 µM), 0.9 × 10^−3^ nds (100 µM), and 1.0 × 10^−3^ nds (150 µM) from the J6/JFH1 reference strain. Divergence between day 4 and day 9 samples was also estimated and found to increase with increasing drug concentrations; divergence between day 4 and 9 was 1.3 × 10^−3^ nds for the NTC, compared to 1.3 × 10^−3^ nds (20 µM), 1.6 × 10^−3^ nds (100 µM), and 2.1 × 10^−3^ nds (150 µM). These observations link the effect of RBV to an accelerated mutation rate of HCV by revealing a steady accumulation of mutations despite inhibition of virus spread. Continuous accumulation of mutations also suggests an intact viral RNA replication.

### Mutations accumulate as single nucleotide changes in individual supernatant viruses

**Figure 2:**
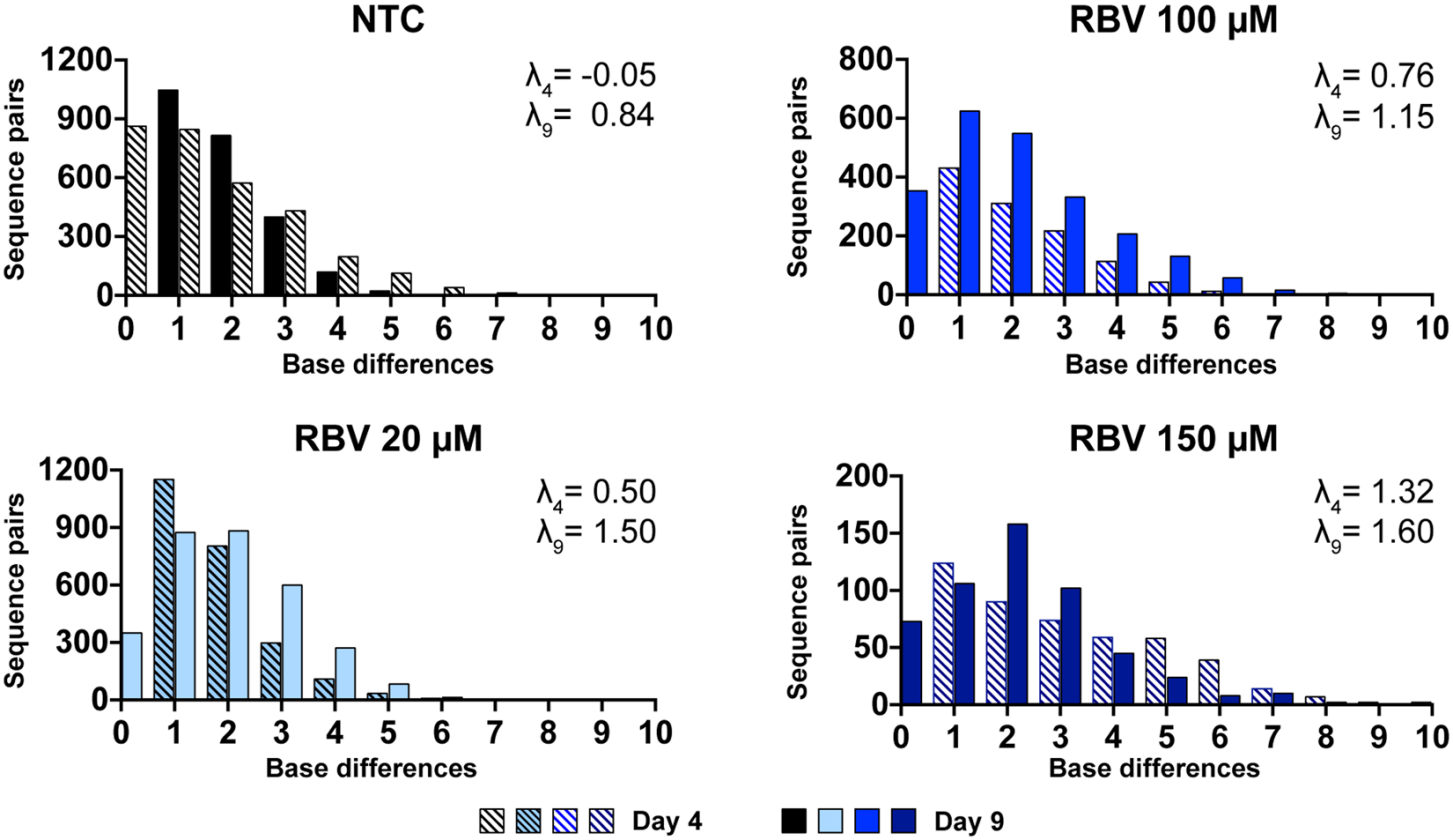
Hamming distances in HCV sequences obtained from supernatants of RBV treated and non-treated J6/JFH1 cell cultures. Graphs show the distribution of number of base differences per sequence pairs, at day 4 (banded bars) and day 9 (solid bars) for each sample. Colors represent different RBV dilutions matched to the values in Fig. 1. The shown lambda values were calculated by fitting the datasets to a Poisson distribution. Sample sizes for each sample are found in Table 1.

In order to understand the observed mutational pattern, we next investigated Hamming distances. Hamming distance plots were obtained by counting the number of nucleotide differences between pairs of sequences, plotting them against the number of sequence-pairs, and fitting them to a Poisson distribution. The lambda (λ) value of the Poisson curve reflects the average number of differences between pairs of sequences: greater lambda values indicate populations with higher mean number of differences (Fig. 2). Treatment with RBV resulted in a right shift of Hamming distance curves, with average distances increasing by 1-3 nucleotide changes per sequence pair with the concentration of RBV. Lambda values on day 9 were 0.84 in the NTC compared to 1.50 (20 µM), 1.15 (100 µM), and 1.60 (150 µM). We did not observe occurrence of sequences with very high number of mutations (more than 10). The increase in nucleotide diversity was driven by induction of single mutations in individual virus genomes.

### Ribavirin tri-phosphate and mono-phosphate have effects comparable to that of RBV on J6/JFH1 infection

**Figure 3:**
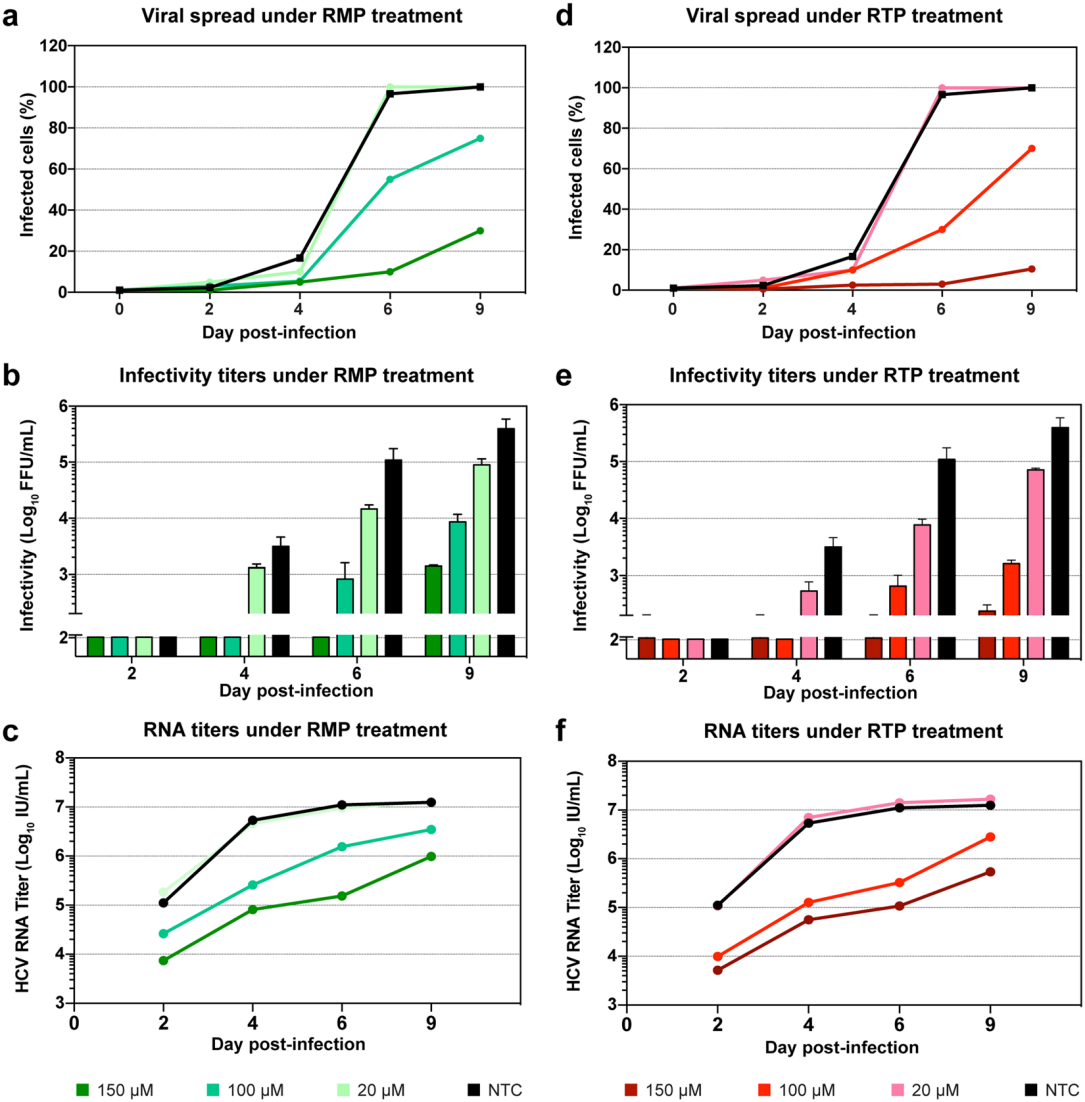
Treatment of HCV J6/JFH1 infected Huh-7.5 cells with RMP and RTP. Representative charts depicting one of two sets of independent experiments (for the second set of experiments see Supplementary Fig. 1). (**a** and **d**) Infection spread of J6/JFH1 virus estimated by immunostaining of NS5A in samples treated with RMP and RTP, respectively. Data points express average of 1–3 determinations. (**b** and **e**) Viral infectivity of filtered supernatants obtained from samples treated with RMP and RTP, respectively. Data points represent average of at least 3 determinations, error bars indicate SD. (**c** and **f**) HCV RNA titers of viral supernatants from treated and non-treated samples, data points represent averages of at least 2 measurements. The colors explanation is visible at the bottom of the figure.

RBV is a pro-drug, requiring intra-cellular phosphorylation for activation. Several cellular enzymes are responsible for the phosphorylation process, but the initial phosphorylation of RBV to RBV monophosphate (RMP) by the cellular adenosine kinase (ADK) is considered a critical limiting step for the formation of both RMP and RBV triphosphate (RTP). Recent findings^41^ suggested that Huh7 cell lines might have impaired expression of ADK, reducing the sensitivity of these cells to RBV treatment. To investigate the effect of RBV phosphorylation in our cell-culture system, we treated infected Huh7.5 cells with RMP and RTP. When tested against J6/JFH1 in a dose-response assay, these compounds showed IC_50_ of 277 µM for RMP and 330 µM for RTP, similar to that of RBV (Fig. 1a). Both drugs showed higher cytotoxicity than RBV, with LC_50_ of 4.4 mM (RMP) and 2.0 mM (RTP) (Fig. 1b), however the assay showed more than 90% live cells at concentrations of up to 300 µM, thus allowing us to perform RMP and RTP treatment using the same range of concentrations as for RBV. Infections and treatment were conducted in the same fashion as done with RBV. The effects of RMP and RTP on virus spread and infectivity were very similar to what had been observed with RBV (Fig. 3 and Supplementary Fig. 1). Both drugs were less effective at fully inhibiting viral spread, even at the highest concentration. At day 9, infection had spread to 30% and 10% of the entire cell culture when the cultures were treated with RMP 150 µM and RTP 150 µM, respectively. Viral infectivity also decreased up to 3 logs as observed with RBV treatment (Fig. 3b and e). In a similar pattern to what was seen with RBV treatment, supernatant virus RNA titers declined only 1–2 logs compared to NTC, in a concentration-dependent manner, again showing a more limited effect of treatment on HCV RNA production in comparison to viral spread and infectivity (Fig. 3c and Fig. 3f). At day 9, viral infectivity reduction was 2.2- and 2.3-fold higher than the decrease in RNA titers for 150 µM RMP and RTP, respectively. The difference in reduction between RNA and infectivity is similarly reflected in the significant reduction in specific infectivity of samples treated with both RMP and RTP at 100 µM and 150 µM (Supplementary Fig. 2). Overall, inhibition of HCV by RMP and RTP was concentration-dependent and showed efficacy comparable to RBV, indicating that phosphorylation by ADK is not a limiting factor in our system and supporting a model in which RBV acts through its tri-phosphorylated form.

### Ribavirin associated transitions (G-to-A and C-to-U) increase more than non-ribavirin associated transitions (A-to-G and U-to-C) with increasing antiviral activity

To investigate whether mutations generated under treatment were ribavirin-associated transitions (G-to-A and C-to-U), we next analyzed the number and type of mutations detected. All sequences from day 9 samples treated with RBV, RMP, and RTP were compared to the J6/JFH1 reference strain. To calculate frequencies of base changes, the number of mutations was normalized to the frequency of each nucleotide in the reference sequence and the number of sequences in the alignment. Table 1 reports the numbers, kinds, and significances of mutations in treated and non-treated samples. In samples treated with RBV the increase in G-to-A was significantly accelerated; the frequency of G-to-A changes in the NTC was 0.036% compared to 0.032% (20 µM RBV; not significant), 0.088% (100 µM RBV; p = 0.01) and 0.126% (150 µM RBV; p < 0.0001). The frequency of C-to-U in the RBV samples (0.066%, 0.077%, and 0.068% for 20 µM, 100 µM, and 150 µM, respectively) was higher than that in the NTC (0.045%) although the difference was not statistically significant. Among cultures treated with RMP, all samples showed a significant increase of G-to-A transitions, which raised from 0.036% (NTC) to 0.076% (20 µM; p = 0.01), 0.078% (100 µM; p = 0.03), and 0.150% (150 µM; p < 0.0001). C-to-U transitions were increased at all RMP concentrations, compared to the NTC, although this increase was not statistically significant. Lastly, samples treated with RTP showed a marked increase in C-to-U transitions compared to the NTC; frequencies were 0.045% (NTC) compared to 0.091% (20 µM; not significant), 0.145% (100 µM; p = 0.03) and 0.265% (150 µM RBV; p < 0.0001). On the other hand, G-to-A changes and other non-ribavirin associated mutations did not increase significantly in these samples.

**Figure 4:**
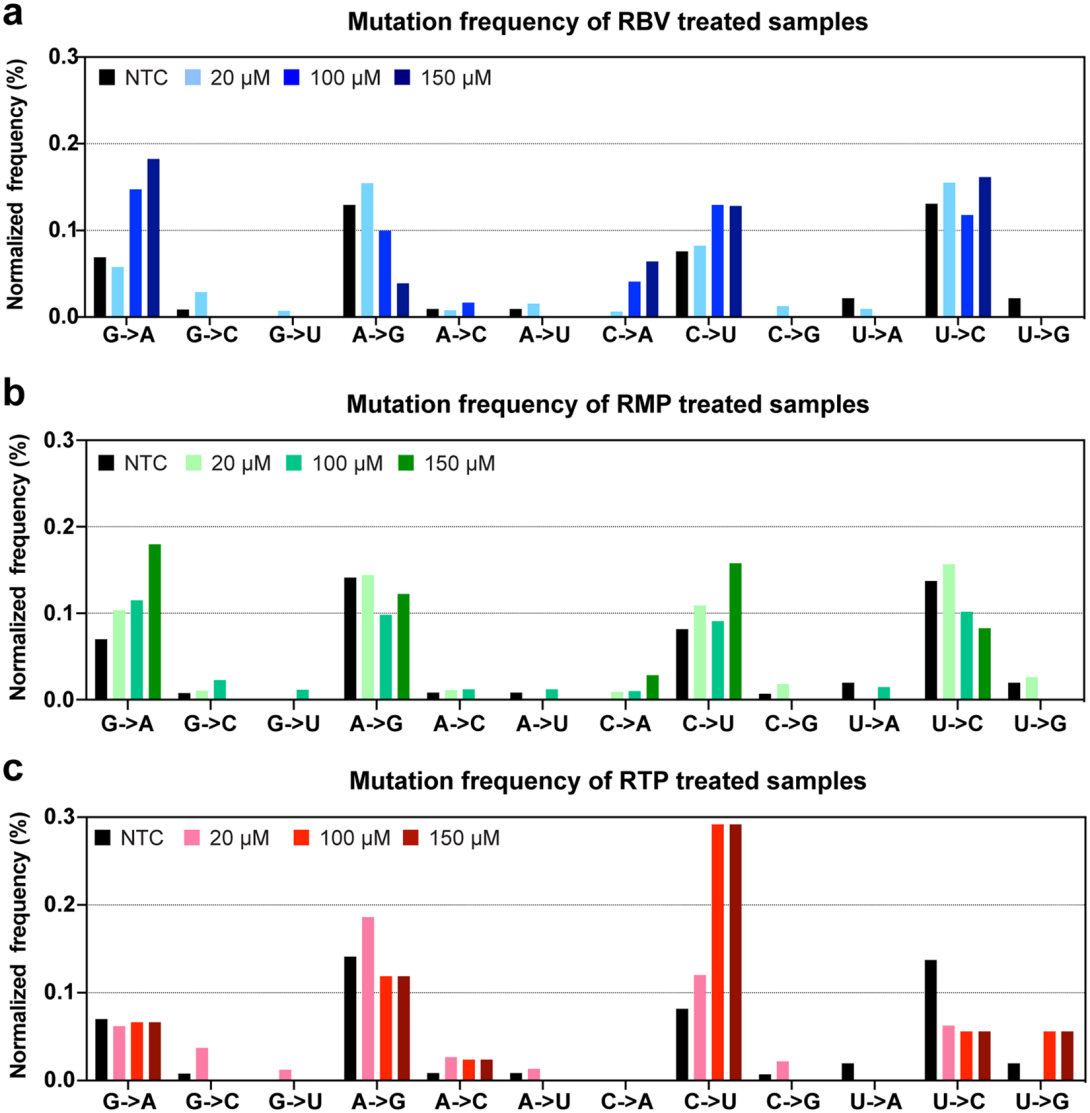
Mutations frequencies in unique HCV sequences obtained from supernatants of treated and non-treated J6/JFH1 cell cultures. All nucleotide substitutions identified in unique sequences were scored and normalized to the number of each nucleotide in the baseline reference sequence. (**a**) Mutation frequencies from day 9 samples treated with RBV. (**b**) Mutation frequencies from day 9 samples treated with RMP. (**c**) Mutation frequencies from day 9 samples treated with RTP. Drug concentrations are indicated by color legends in each panel. Bars represent overall frequencies calculated from unique sequences. Sample sizes: n = 37 (NTC); n = 115 (RBV); n = 76 (RMP), n = 61 (RTP).

To verify that the observed increase in RBV-associated mutations was driven by treatment and was not the result of co-selection or sequence resampling, we excluded all identical sequences and repeated the analyses on unique sequences only (Fig. 4). Notwithstanding the lower overall number of sequences included in the analysis (sample sizes were 37 for NTC, 115 for RBV, 76 for RMP, and 61 for RTP), the resulting mutation frequencies were similar to those observed in the unfiltered dataset, showing significant peaks for G-to-A and C-to-U transitions. In some instances, the relative increase of RBV-associated mutations in treated samples versus NTC was more pronounced when identical sequences were filtered out, whereas non-RBV-associated mutations were reduced. Samples treated with 150 µM RMP, for instance, showed a significant increase in C-to-U transitions (from 0.096% to 0.157%; p < 0.0001) compared to the NTC (0.045%), while differences at lower concentrations remained non-significant.

Overall, treatment with phosphorylated and non-phosphorylated forms of RBV resulted in a marked increase in G-to-A and C-to-U transitions. The effect was, at least partially, concentration-dependent and resulted in 2- to 4-fold increase in the frequency of RBV-associated transitions (Table 1 and Fig. 4). In addition, the mutation profiles produced by RBV, RMP, and RTP were comparable, again supporting a model in which RBV acts through its tri-phosphorylated form. These observations confirm that the increase in virus population diversity when treating with RBV and its phosphorylated forms is driven by an increase in the frequency of RBV-associated mutations. Subsequently, we investigated whether mutations were grouped at specific sites in the HCV NS5B-polymerase region.

### Uniform accumulation of ribavirin-associated mutations in the HCV genome

**Figure 5:**
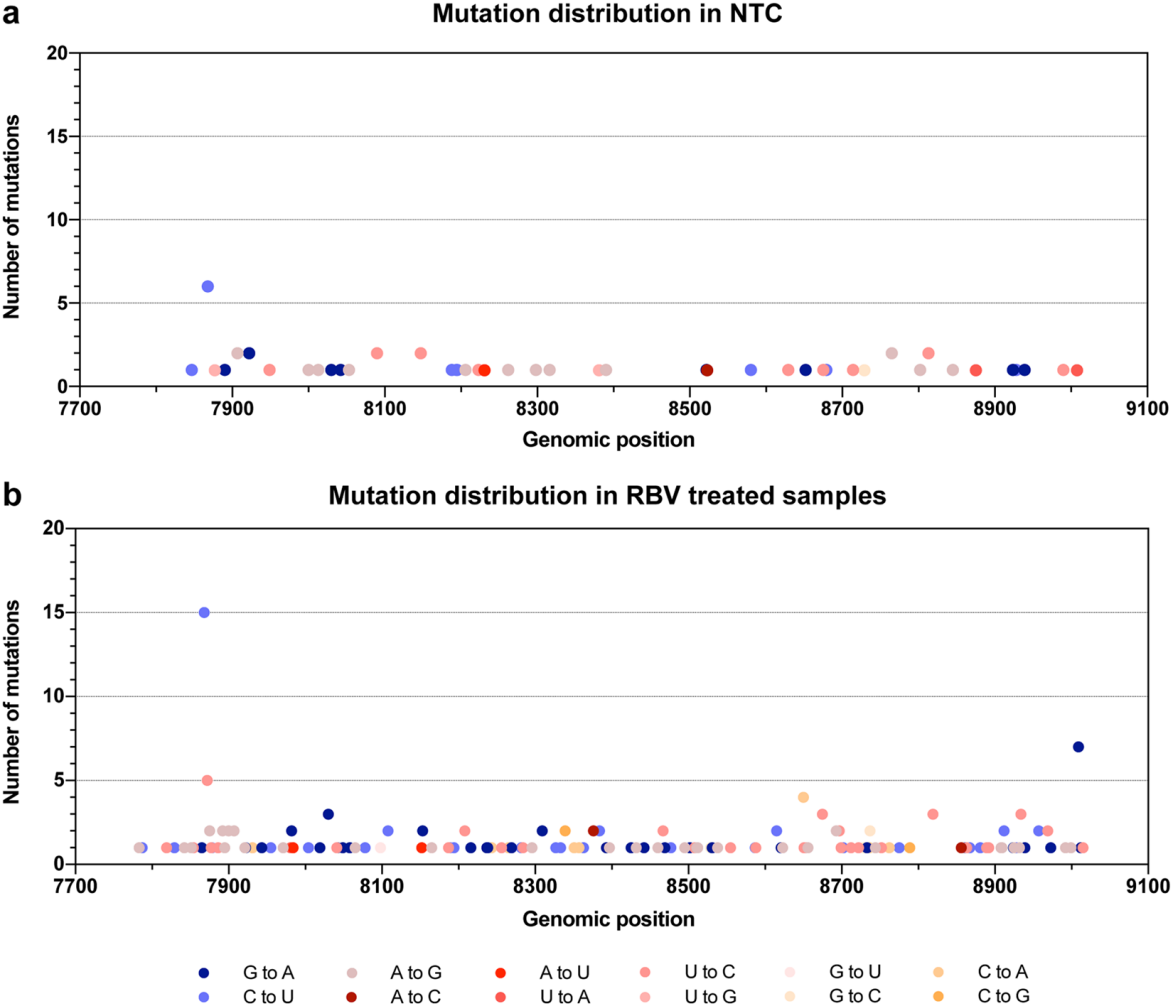
Location of nucleotide substitutions along the analyzed HCV NS5B region of J6/JFH1 in RBV treated and non-treated samples. The graphs depict the position and frequency of each substitution identified in (**a**) NTC (n = 78) and (**b**) RBV-treated samples (n = 177). Colors define the kind of substitution, with blue tones representing RBV-associated mutations and red tones representing other mutations. Positions are relative to H77 reference HCV genome (Accession number: AF009606).

Figure 5 illustrates the numbers and kinds of mutations in reference to the analyzed J6/JFH1 NS5B sequence for non-treated controls (Fig. 5a) and samples treated with RBV at day 9 (Fig. 5b). In the 78 analyzed sequences from untreated controls we identified 58 total mutations, of which 40 were unique mutations. Mutations occurring in more than two sequences were equally distributed between RBV-associated and non-associated mutations (10 were A-to-G or U-to-C vs 8 G-to-A or C-to-U). A total of 190 mutations were identified in the 177 analyzed sequences from treated samples, of which most (107 nucleotide changes) were unique. Most RBV-associated mutations were also unique (44 of 76 mutations). Similarly to NTCs, mutations occurring in more than two sequences were not predominantly RBV-associated mutations (32 were A-to-G or U-to-C vs 41 G-to-A or C-to-U). Both RBV-associated and non-associated mutations appeared to be overall randomly distributed along the analyzed region. Only 2 sites had a relatively high mutation frequency in treated samples, these were positions 7933 and 9074 with 15 and 7 mutations, respectively (corresponding to positions 7868 and 9009 in the H77 reference genome). Position 7933 was also observed to have a higher mutation frequency in NTCs (6 mutations; Fig. 5a) and in experiments with RTP and RMP (7 and 5 mutations, respectively (data not shown)). The mutation was a C-to-U and did not confer changes in the amino acid composition. Currently, it is unclear why this position was prone to mutate; it has not previously been associated with RBV exposure and since it was synonymous and identified also in NTCs, it is unlikely to directly confer resistance to RBV. Overall, RBV-associated mutations were mostly unique and uniformly distributed in the NS5B region.

### Generation of Huh7.5 cell-line overexpressing ADK

**Figure 6:**
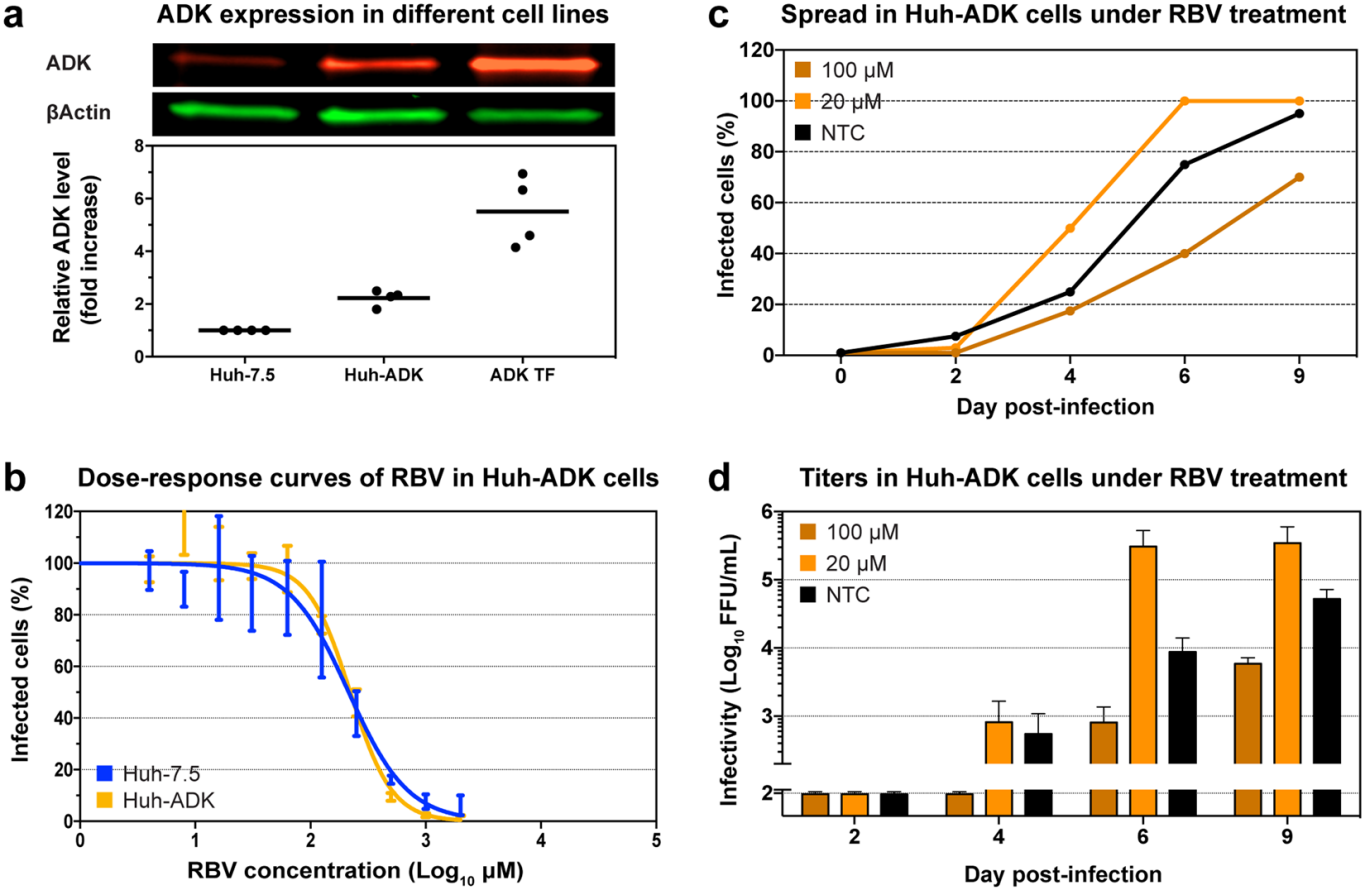
Efficacy of RBV against HCV J6/JFH1 in Huh-ADK cells. (**a**) Fluorescent western blot analysis of the expression of ADK in different cell lines. The red and green pictures display a representative western blot of ADK and βActin stained simultaneously with different antibodies. The chart depicts normalized quantification of ADK expression, relative to Huh7.5 stock cell line. Lines represent the mean value of 4 independent quantifications. Huh-ADK: stably expressing ADK; ADK-TF: Huh7.5 transiently transfected with ADK expression plasmid. See Supplementary Fig. 3 for the full-length blots. (**b**) Dose-response curves of RBV performed in Huh7.5 and Huh-ADK cells. Values were obtained from triplicate determinations for each dilution and normalized to non-treated controls. Curve fitting was performed with 0%-100% constraints, error bars represent SD. (**c**,**d**) Representative graph from one of two sets of independent experiments on Huh-ADK cells (for the second set of experiments see Supplementary Fig. 1). (**c**) Infection spread of HCV J6/JFH1 virus under RBV treatment, monitored by immunostaining of NS5A. Data points represent average of 2 determinations. (**d**) Viral infectivity of filtered supernatants obtained from infected Huh-ADK cells treated with RBV. Data points represent average of 2 determinations, error bars represent SD.

To further assess whether reduced ADK expression in our cell system could explain the observed high IC_50_ for J6/JFH1 infections, we produced a derivative cell line of Huh7.5 (Huh-ADK) stably transfected with a plasmid expressing human ADK. Intracellular levels of ADK were determined by quantitative fluorescent western blot in four independent experiments (Fig. 6a). The novel Huh-ADK cell line showed a significant 2.2-fold increase in ADK expression (p < 0.05, paired t-test), while transiently transfected cells displayed a 6-fold increase 48 hours post-transfection (p < 0.05, paired t-test), compared to the original Huh7.5 cell line. Thus, we generated a stably transfected hepatic cell line overexpressing the ADK enzyme, to be used in comparative studies of RBV efficacy in cell-culture.

### Overexpression of ADK does not affect ribavirin treatment outcome

To assess whether ADK overexpression in Huh-ADK would improve the efficacy of RBV against J6/JFH1 infections, we performed a 48 hours dose-response assay using Huh7.5 and Huh-ADK cells in parallel (Fig. 6b). The dose-response curves obtained with these two cell lines were very similar, yielding IC_50_ of 215 µM and 221 µM for Huh7.5 and Huh-ADK, respectively. Subsequently, Huh-ADK cells were infected with J6/JFH1 and treated with RBV at 20 and 100 µM, using the same protocol as used for Huh7.5 cells. The antiviral effect of 100 µM RBV on J6/JFH1 in Huh-ADK cells was very similar to what was observed in Huh7.5 cells, showing delay in viral spread, reduced HCV infectivity, and lower RNA titers (Fig. 6c,d, and data not shown). We sequenced HCV from cell supernatant treated with RBV 20 μM and RBV 100 μM, and observed concentration-dependent enrichment of virus diversity in treated samples, at a similar level as in Huh7.5 cells (Table 1). Accumulation of RBV-associated transitions in J6/JFH1 cultured in Huh-ADK cells was comparable to findings in Huh7.5 cells. These results confirm that ADK expression in our Huh7.5 cell-culture system is not a limiting factor for RBV sensitivity.

## Discussion

Studies of the effect of RBV in HCV infectious cell culture models have been limited in scope^28,29,42^. *In vitro* models have the advantage over clinical studies of controlling for host and virus variability. Furthermore, they recapitulate the complete viral infectious cycle compared to replicon systems, which primarily cover RNA replication^16^. We therefore used a well-established HCV cell culture model^31^ to investigate the effect of RBV on spread, infectivity, RNA production, and mutational spectrum of released viruses.

Our results show that high concentrations of RBV (150 μM) can fully inhibit HCV spread and infectivity in cell culture, while having reduced effect on supernatant virus RNA titers. In a similar *in vitro* assay, our group has studied the effect of the DAA Sofosbuvir (a chain-terminator nucleotide analog) and found it to be associated with a sharp decrease in HCV RNA titers along with inhibition of spread and infectivity^43^, indicating that inhibition of RNA replication can lead to measurable reduction in supernatant HCV genome titers. Our observations of relatively small changes in RNA titers, despite large drops in viral infectivity at the highest drug concentrations, suggest that the biological effect of RBV is mediated through post-transcriptional rather than replicative inhibition. To our knowledge, this is the first time such a dichotomic effect of RBV has been reported for HCV infection, and is an indication that RBV acts through an increased mutagenesis mode of action. These findings are supported by our analysis of the mutational pattern of released viruses, showing that RBV and its phosphorylated forms increase the overall diversity of the viral population in a concentration-dependent manner.

Studies analyzing plasma virus from patients undergoing RBV mono-therapy, have found an increase in the overall complexity of the virus population^24,25^, while others have not^26^. Diversity is a very crude measure of the polymorphisms in a viral population, and can be affected by selection and migration, as well as viral factors including fitness and structural RNA constrains. A possible explanation for these conflicting results could be that these studies compared viruses across different genotypes and infectious stages, so that overlaying confounding factors masked the diversity signal. By studying the evolution of a relatively homogeneous virus inoculum in a controlled environment, *in vitro* cell-culture studies minimize the significance of these factors, thus allowing the detection of small changes in the population sequence space.

We found that RBV-associated transitions (G-to-A and C-to-U) were enriched in HCV genomes from treated cultures. Mutations accumulated in a concentration-dependent way and at a higher frequency (2–4 fold) than non-RBV associated transitions (A-to-G and U-to-C). These results are in good agreement with findings from *in vivo* studies, reporting increase in G-to-A and C-to-U transitions^24,25^. We observed comparable, but not identical mutation profiles in samples treated with RBV, RMP, and RTP. Rather than representing functional differences between the drugs, we believe this is attributable to the relatively limited sample size and number of mutations detected. A recent *in vitro* analysis of RBV effect on HCV also reported comparable increased mutation rates^28^, however, the system used in that study led to cure of HCV infected cells with RBV monotherapy, an event seldom observed both *in vivo* and *in vitro*. Overall, our findings indicate that our cell-culture system can accurately recapitulate the effect of RBV observed in patients and further support a mutagenic mechanism of action for RBV.

We found that mutations were most often unique and synonymous. The genomes in the cell supernatant viruses went from having on average 0–1 mutations (compared to the J6/JFH1 reference) to having an average of 1–3 mutations. It should be noted that any selection pressure on the virus most likely will increase the mutational spectrum, however, the detected mutations were specific to RBV (G-to-A and C-to-U) and increased in frequency despite strong antiviral pressure, which normally would be associated with loss of diversity. We sequenced supernatant culture viruses, which may over-represent functional genomes selected for infectivity or packaging capacity, possibly limiting the detection of the mutagenic potential of RBV. Thus, the mutational ability of RBV may be greater than what we report in here. Also, for the first time we examined the distribution of mutations throughout the NS5B region, revealing that they were overall evenly spread. This suggests that the observed mutations did not represent drug escape mutations, but rather random substitutions induced by RBV treatment.

The concentration of RBV in plasma in treated patients has been estimated to be 10–20 µM, while the concentration in hepatocytes is unknown. We studied a range of concentrations of RBV from 20 µM t*o* 150 µM. The antiviral effect below 100 µM was subtle. These observations are in good agreement with clinical data on RBV, as mono-therapy has little and only transient effect on plasma HCV RNA levels^1^.

Recent studies have proposed that Huh-7 cells have a reduced expression of cellular adenosine kinase (ADK)^41^, a critical enzyme for phosphorylation of RBV into its active forms. We wanted to know if ADK expression would affect the potency of RBV in our culture system, which is based on Huh7.5 cells. We therefore developed a Huh7.5 cell line that stably over-expresses ADK. ADK over-expression did not affect the potency or mutagenic action of RBV in our culture model. These observations were further supported by experiments investigating the effect of phosphorylated forms of RBV. The mono- and tri-phosphorylated forms of RBV showed effects comparable to RBV in inhibiting virus spread and infectivity, and were as effective in inducing RBV-associated mutations. We thus conclude that ADK activity is not a limiting factor on RBV activity in our infectious cell culture system. It should be noted that Mori and coworkers^41^ used Huh-7 cells for their experiments, which could possess different native ADK levels compared to Huh7.5 cells. In addition, the similar activities observed for RBV, RMP, and RTP, support a model of action of RBV through its tri-phosphate form RTP, which is the common phosphorylation end point of both RBV and RMP.

Overall, our data support a mechanism of action of RBV as a mutagen, mediated by its tri-phosphate form, acting by increasing the frequency of RBV-associated transitions (G-to-A and C-to-U) in the viral population. Lethal mutagenesis should be explored as an antiviral strategy for the development of new broadly acting antivirals, which are urgently needed in the modern world with increased risk of emerging or re-emerging diseases, in particular outbreaks by mutable RNA viruses, that require fast intervention.

## Materials and Methods

### Cell culture and infections

The human hepatoma derived cell line Huh7.5^32^ was grown in Dulbecco’s Modified Eagle Medium (DMEM, Gibco) supplemented with 10% fetal calf serum, 100 µg/mL streptomycin, and 100 u/mL penicillin (Gibco). The Huh-ADK cell line generated in the present study (see below) was maintained in the same medium as Huh7.5, supplemented with 250 µg/mL Hygromycin-B (InvivoGen). For infections, 1.8 × 10^6^ naïve cells were plated on 10 cm dishes and infected at MOI 0.0005 with culture-derived sterile-filtered J6/JFH1^31^ supernatant 24 hours post-seeding. To minimize carryover of input virus, cells were washed twice with PBS 4 hours after inoculation, briefly treated with 10% trypsin-EDTA (Gibco), and washed again with PBS. Cells were then incubated overnight in complete medium. Infected cells were split 24 hours post-infection, plated in 25 mL flasks at 4 × 10^5^ cells per flask, and subjected to treatment. For treated cultures, medium containing RBV or its related forms was added at each cell split.

### Generation of Huh-ADK cell line

We produced a derivative cell line of Huh7.5 (Huh-ADK) stably transfected with a plasmid expressing human ADK. The commercial expression plasmid pCMV-ADK (HG13149-G-N, Sino Biologicals) encoding the cDNA of human ADK (accession number BC003568) and the Hygromycin resistance selection marker was transfected into Huh7.5 cells. Selection of stably transfected cells was started 48 hours post-transfection by culturing cells with 500 µM Hygromycin-B. After 2 weeks of selection, surviving cells were pooled and maintained in 250 µM Hygromycin-B for subsequent experiments. Overexpression of ADK was verified by quantitative western blot analysis.

### Monitoring of infection spread and viral titers

Infected cells were split and viral supernatants collected every 2 to 3 days. Viral spread was monitored by immunostaining of cells, plated on microscopy slides, with primary mouse antibody against HCV NS5A protein (9E10)^31^ and secondary goat anti-mouse antibody conjugated to AlexaFluor 488 (ThermoFisher). Viral infectivity was determined using poly-D-lysine-coated 96-well plates (Nunc) seeded with 6 × 10^3^ Huh7.5 cells per well, as previously described^44,45^. Cells were inoculated in triplicate with 100 µL of sample dilutions ~24 hours post seeding. Fixation with ice-cold methanol and overnight immunostaining were performed 48 hours after infection, using the 9E10 primary antibody. Secondary staining was carried out with anti-mouse antibody linked to HRP enzyme (GE Healthcare Amersham), followed by 30 minutes incubation with DAB substrate (DAKO) to produce colored precipitate in positive cells. The number of FFU was determined on an ImmunoSpot Series 5 UV Analyzer (CTL Europe GmbH) with customized software, as previously described^45^. In the determination of viral infectivity of treated samples, carryover drug present in the supernatant could affect the FFU readout. However, given that samples were diluted at least 2 to 10 times for this assay, and that drug concentrations below 100 µM had marginal effect of J6/JFH1 spread, we estimate this effect to be negligible for the purpose of this study.

### Antiviral Drug Treatment

All compounds used were synthesized by ACME Bioscience (Palo Alto, USA). Lyophilized compounds were re-suspended in PBS upon receipt to a final concentration of 100 mM, aliquoted, and stored at −20 °C. Working dilutions were prepared fresh before use by serially diluting stock solutions in complete DMEM at the desired concentrations. In treatment experiments, drug dilutions were applied to infected cells one day post-infection and every 2–3 days thereafter, upon cell splitting.

### Dose-response and cell viability assays

Cells were plated at 6 × 10^3^ cells per well on poly-d-lysine-coated 96-well plates (Nunc). To determine the dose-response curve of RBV and related compounds, cells were infected ~24 hours post seeding with J6/JFH1 stock virus at high MOI, then treated with triplicate serial dilutions of drugs 24 hours post-infection. Cell were fixed in methanol 72 hours post-infection, immunostained with primary 9E10 antibody, secondary anti-mouse antibody linked to HRP enzyme (GE Healthcare Amersham), and colored with DAB substrate (DAKO). Infected cells were counted individually using ImmunoSpot Series 5 Analyzer (CTL Europe GmbH) with customized software, as previously described^44,46^. To assess cell viability under treatment, naïve cells were treated in triplicate with serial dilutions of drugs for 48 hours, and the percentage of live cells compared to untreated controls was determined using CellTiter 96 AQueous One Solution cell proliferation assay (Promega), following manufacturer’s instructions.

### Fluorescent western blot

Cell lysates were prepared using RIPA buffer (ThermoFisher) supplemented with protease inhibitors, following manufacturer recommendations. Briefly, cells were washed twice with cold PBS, lysed in cold RIPA buffer for 30 minutes, centrifuged for 15 minutes at maximum speed at 4 °C, supernatants collected and stored at −20 °C for subsequent analysis. Total protein concentration was determined on a Qubit fluorometer using the Qubit Protein Assay Kit (ThermoFisher), according to recommendations. PAGE was performed using Mini-PROTEAN TGX 4–20% gels (Biorad), and separated proteins were blotted on Immun-Blot Low Fluorescence PVDF membranes (Biorad). Membrane blocking was performed in Rockland blocking buffer for fluorescent western blotting (Rockland Immunochemicals) overnight, followed by primary staining in the same buffer using rabbit-anti-ADK antibody at 1:200 (ab38010, AbCam) and mouse-anti-βActin antibody at 1:1000 (sc47778, Santa Cruz Biotechnology). Secondary staining was carried out in PBS using goat-anti-rabbit antibody conjugated to AlexaFluorPlus 647 and goat-anti-mouse antibody conjugated to AlexaFluorPlus 555 (ThermoFisher). Imaging was obtained with a Chemidoc MP (Biorad) equipped for multicolor western-blot. Protein band quantification was calculated using ImageLab 6.0 (BioRad) using β-Actin bands for normalization (see Supplementary Fig. 3).

### HCV RNA extraction and sequencing

HCV RNA was extracted from 50 µL of cell culture supernatant as described previously^36^. Viral RNA was transcribed into cDNA as previously described using JB9470R_JFH1 (reverse) 5′-CTATGGAGTGTACCTAGTGTGTGC-3′^47^ and a 1245 bp region of the NS5B gene was amplified by nested PCR using primers NS5B_F1 (forward) 5′-TTGCTCCGAGGAGGACGATAC-3′ and JB9470R_JFH1 (reverse) for first round PCR, and NS5B_F2 (forward) 5′-GTAACTCGCTGTTGCGATAC-3′ and NS5B_R2 (reverse) 5′-CGGTGAACCAACTGGATAAGTC-3′ for second round PCR. Amplification was carried out using Platinum Taq DNA polymerase high fidelity (Invitrogen) following manufacturer recommendations and annealing temperature of 55 °C in both rounds of PCR. Molecular cloning of PCR products was performed using the TOPO TA cloning kit (ThermoFisher) according to manufacturer instructions. Plasmids were extracted with the Nucleospin 8 kit (Macherey-Nagel) and bi-directionally sequenced using 4 primers (Macrogen Europe).

### Viral RNA titers

For determination of HCV RNA titers in culture supernatant, RNA was extracted from 200 µL supernatant using the Total Nucleic Acid Isolation Kit (Roche Applied Science); titers were determined by TaqMan real-time PCR as previously described^47^.

### Genetic analyses

Sequences were assembled using the Lasergene software (DNASTAR). Sequences were manually checked for errors and aligned to the J6/JFH1 reference sequence using the ClustalW algorithm integrated in Bioedit 7.2.5 software^48^. In total, 1096 individual sequences were produced and analyzed. Of these, only two contained stop codons in the NS5B reading frame and three contained single nucleotide deletions (which were not counted in the mutation analyses). Sequence diversity and divergence from the J6/JFH1 reference sequence were estimated using average pairwise distance (p-distance) corrected by the Jukes and Cantor model (JC), as described by Nei and Kumar, using MEGA 7.0 software (MEGA7: Molecular Evolutionary Genetics Analysis version 7.0 for bigger datasets^49^). This distance is the proportion of nucleotides that are different between two sequences, calculated dividing the number of nucleotide changes by the total number of nucleotides and corrected using the Jukes-Cantor nucleotide substitution model. Briefly, sequences from each sample were compared among themselves (diversity) or to the reference J6/JFH1 consensus sequence (divergence) to obtain average p-distance and SD that describe diversity and divergence for each sample. RBV associated mutations (G-to-A and C-to-U) and non-RBV associated mutations (A-to-G and U-to-C) were counted by comparing to the baseline reference sequence, using the program Hypermut^50^, and the percentage was obtained by dividing with the number of sequences in the alignment and normalizing for the number of each particular base (e.g. G if counting G-to-A changes) in the reference sequence, and calculated as percentage. Sequence average number of difference within sets of sequences was calculated by using the DNAsp program 5.10^51^.

### Statistical analysis

Dose-response and cytotoxicity curves were estimated using non-linear regression curve-fitting on baselined and normalized values, with variable slope and constrains at 0% and 100%. Lambda values were obtained by fitting the distribution of Hamming distances to a Poisson curve. Variation significance among sample groups was evaluated using 2way ANOVA with multiple comparisons. The Mann-Whitney U test was used to test differences in mutation frequencies between groups. All statistical analyses were performed using Prism 7 (GraphPad Software).

### Data availability

The Huh-ADK cell line is available upon request and completion of standard Material Transfer Agreement. All sequences generated and analyzed in this study are available from the Genbank repository (Accession numbers: MG890638 - MG891733).

## Electronic supplementary material

Supplementary Figures
