# Supplementary material for: Antiviral Effect of Ribavirin against HCV Associated with Increased Frequency of G-to-A and C-to-U Transitions in Infectious Cell Culture Model
Source: Sci Rep. 2018 Mar 15;8:4619. doi: 10.1038/s41598-018-22620-2 (PMC5854589; doi:10.1038/s41598-018-22620-2)
Supplement: Supplementary file 1 — Supplementary Figures [file 41598_2018_22620_MOESM1_ESM.pdf]

Antiviral Effect of Ribavirin against HCV Associated with Increased Frequency of G-to-A and C-to-U Transitions in Infectious Cell Culture Model

Authors: Andrea Galli, Helene Mens, Judith M. Gottwein, Jan Gerstoft, Jens Bukh

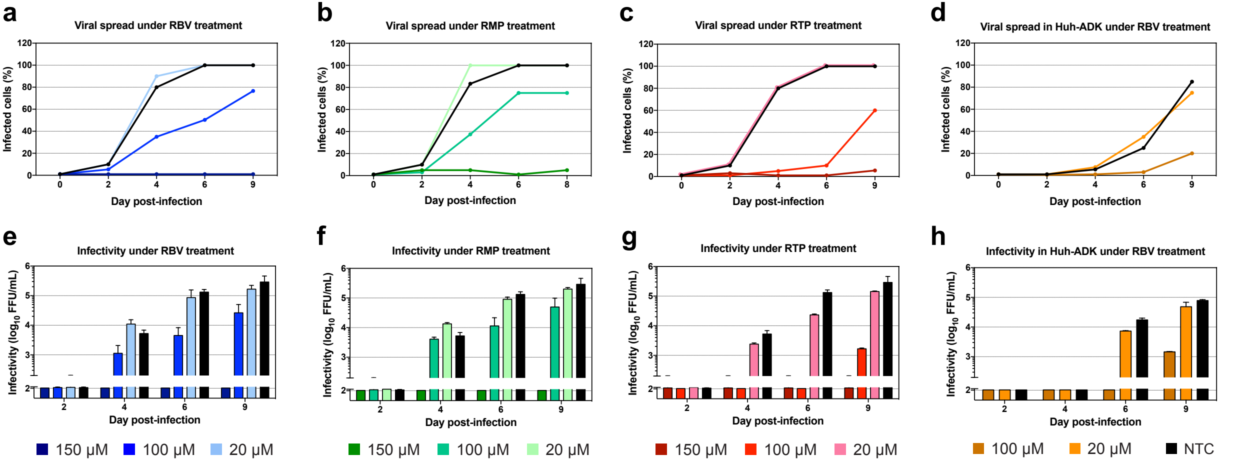

Supplementary Figure 1. Replicate infection experiments. Infection spread of HCV J6/JFH1 virus estimated by immunostaining of NS5A in samples treated with RBV (a), RMP (b), RTP (c), and RBV in Huh-ADK cells (d). Viral infectivity of filtered supernatants obtained from samples treated with RBV (e), RMP (f), RTP (g), and RBV in Huh-ADK cells (h). Graphs are obtained from one of two sets of independent experiments; data points represent the average of 1-3 determinations. Error bars represent standard deviation (SD). The colors explanation is visible at the bottom of the figure.

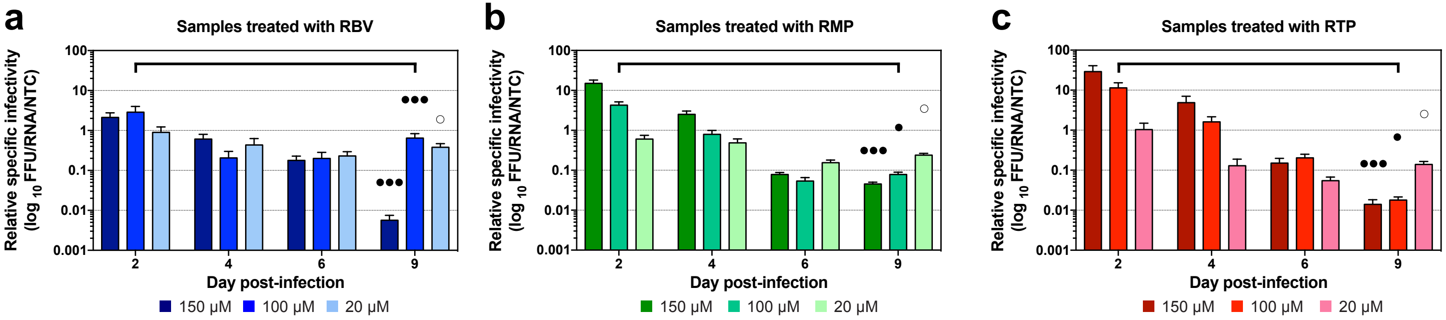

Supplementary Figure 2. Relative specific infectivity of treated samples. Variation over time of relative specific infectivity of samples treated with RBV (a), RMP (b), and RTP (c). Values are calculated as number of FFUs/mL (infectivity) divided by the number of HCV RNA genomes and normalized to NTC. Error bars represent standard deviation. Statistical analysis was conducted between values obtained from days 2 and 9, using 2way ANOVA with multiple comparisons. Determinations were obtained from at least 3 measurements, p-values are indicated by dots: ○: non-significant; ●: $p<0.05$; ●●: $p<0.001$; ●●●: $p<0.0001$

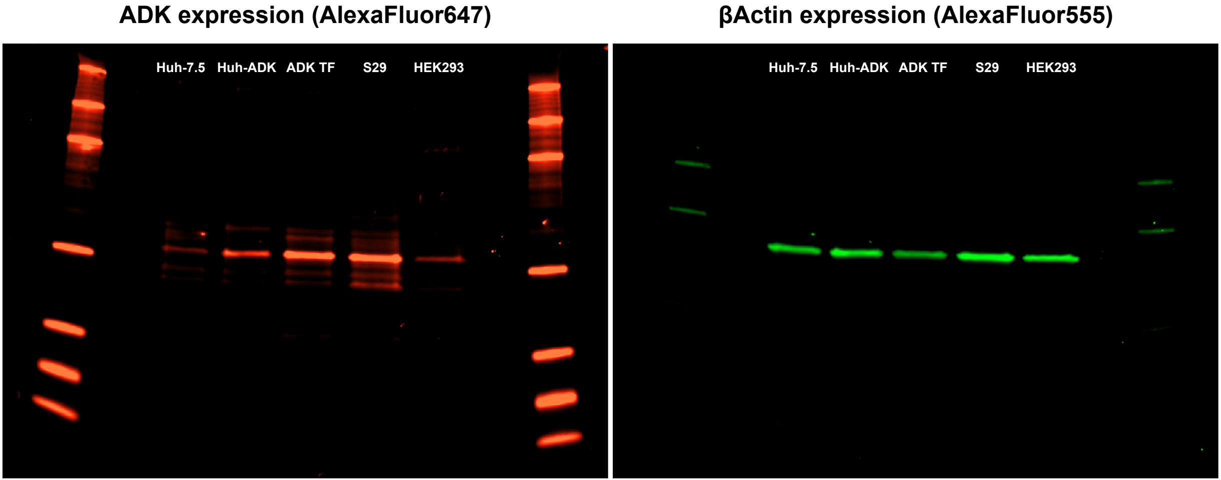

Supplementary Figure 3. Full-length western blot evaluating ADK expression in different cell-lines. Fluorescent western blot analysis of the expression of ADK in different cell lines. The red and green pictures display a representative western blot of ADK and β-Actin stained simultaneously with different antibodies. Huh-ADK: stably expressing ADK; ADK-TF: Huh7.5 transiently transfected with ADK expression plasmid; S29: Huh7.5 derivative lacking CD81 expression; HEK293T: human embryonic kidney cell line 293T .
